# Barber's hair sinus in a female hairdresser: uncommon manifestation of an occupational disease: a case report

**DOI:** 10.1186/1757-1626-1-214

**Published:** 2008-10-06

**Authors:** Christopher Efthimiadis, Christopher Kosmidis, George Anthimidis, Marios Grigoriou, Sofia Levva, Panagiotis Fachantidis, George Psihidis

**Affiliations:** 1 Department of Surgery, Interbalkan European Medical Center, Thessaloniki, Greece; 2 Department of Neurosurgery, Interbalkan European Medical Center, Thessaloniki, Greece

## Abstract

**Background:**

Barber's disease is an acquired occupational disease produced by short customers' hairs that penetrate the interdigital spaces of the hands. The lesion has been reported to occur mostly on the hands of male hairdressers. The purpose of this article is to report a rare case of a female hairdresser who developed a pilonidal sinus in the interdigital web of her non-dominant hand and review the relevant literature.

**Case presentation:**

A 29 year-old Greek female hairdresser underwent surgical excision of the pilonidal sinus and curettage. She was not hospitalised, while prompt resolution of the condition and prevention of recurrence was achieved.

**Conclusion:**

Pilonidal sinus of the interdigital spaces of the hand is a rare and preventable acquired occupational disease. The personal hygiene with thorough removal of any hairs that have penetrated the epidermis during the working day could prevent the formation of the disease. Surgical excision, curettage and primary healing seem to be a safe method of treatment.

## Introduction

Pilonidal sinus is an acquired condition caused by penetration of hair fragments into the skin. The following chronic inflammatory response results in the formation of a sinus and later a cyst. More hairs then become entrapped within the established sinus [1]. Pilonidal sinus usually develops in the sacrococcygeal area or other hair-bearing areas [2]. It has also been described as an occupational hazard in barbers, especially when presented interdigitally [3]. Short customers' hairs that penetrate the supple interdigital skin of the hands produce barber's disease. So far, barber's hair sinus has mostly been reported to occur on the hands of male hairdressers [4].

We present herein a case of a female hairdresser who developed a pilonidal sinus in the third web space of her non-dominant hand.

## Case presentation

A 29-year-old Greek female right-handed hairdresser presented in our outpatient unit with a draining cyst in the palm of her left hand. Nineteen years ago, after accidental drop of strong glue between her middle and ring fingers, a papule had developed in the third web space. She reported that customers' short cut hair often penetrated the third web space skin. She was otherwise healthy and had never suffered from another skin disease. In September of 2003, however, while in pregnancy, she had first reported symptoms consistent with acute inflammation on the palm of her left hand. The papule had developed into sinus, gained connection within the skin and evolved into fistula with hairs protruding in the palm of the left hand. Although the patient had already noticed these alterations, she did not pay much attention to the lesions, as they did not excruciate her.

**Figure 1:**
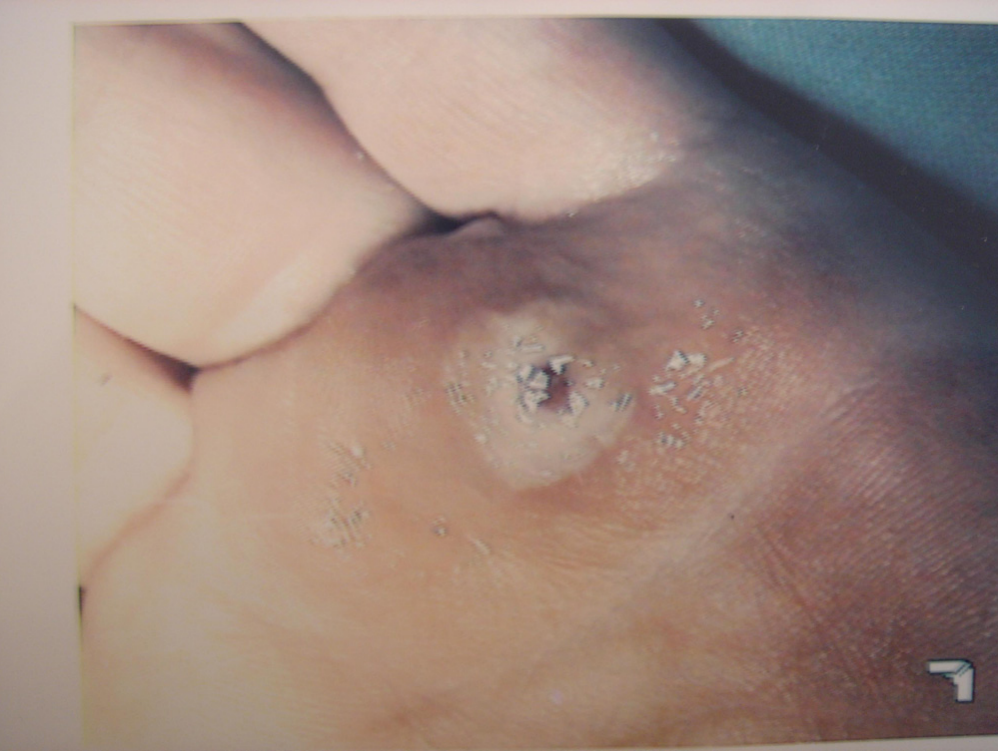
External opening of a hair sinus in the palm of the left hand.

Two years later she visited our outpatient unit with a chronic sinus on the palm of her left hand. The drainage had persisted for two weeks and had not resolved with oral antibiotic therapy. The sinus was 4 cm long, it had two external openings, in the palm of the hand and in the third web space, and the track contained multiple hairs of different colours (Figure 1). Palpation of the interdigital fold provoked discharge of purulent fluid. Examination of the remaining interdigital spaces of both hands and feet did not reveal any pathological lesions. Furthermore, no signs of lymphadenitis occurred.

**Figure 2:**
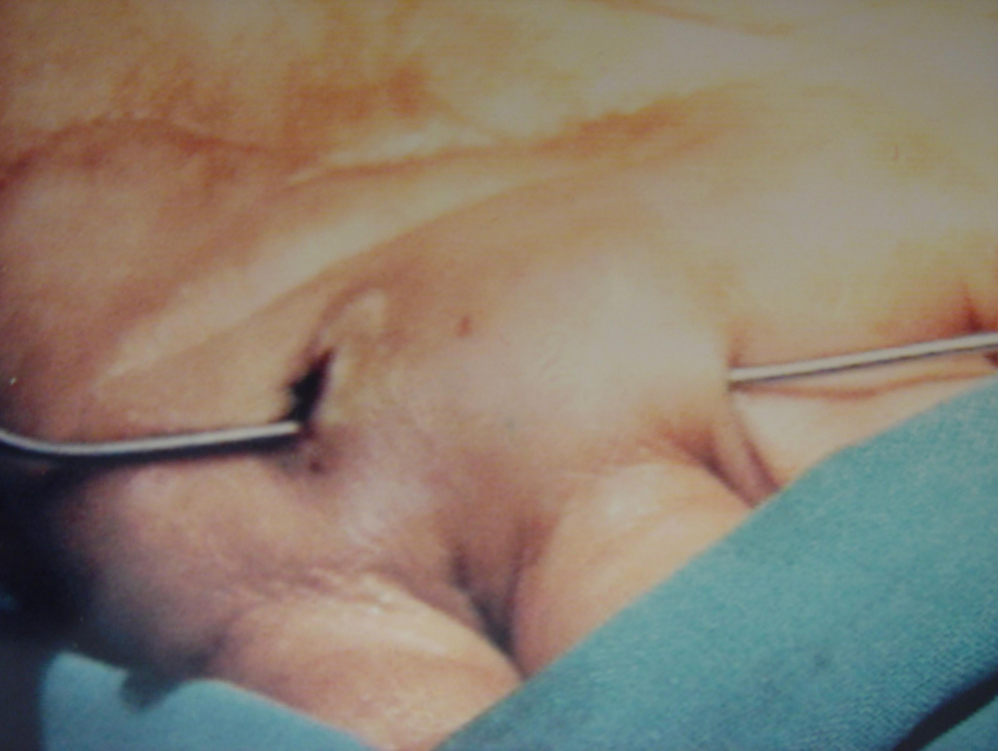
A probe passed along the sinus.

A probe was passed along the sinus (Figure 2). Surgical excision of an area of chronic inflammatory tissue with a sinus tract and multiple visible hairs was carried out under local anesthetic. The drainage and discomfort resolved immediately after the excision. After careful curettage, since all the granular tissue had been removed, the skin was closed by primary intention (two layers). In this way, the functional result could be better and achieved quicker. The patient was discharged the same day and a course of oral antibiotic therapy (amoxycillin-clavulanic acid: 1 g × 2) was administered for 4 days.

**Figure 3:**
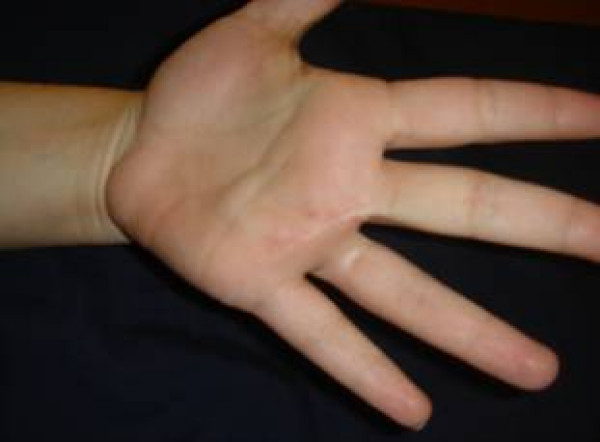
Opening of the sinus tract and excision of the cyst and of multiple visible hairs.

Within 1 week the wound healed well (Figure 3). Histology revealed epithelial-lined tract with multiple embedded hairs and surrounding foreign body reaction granuloma. There has been no recurrence over the next 12 months and she continues working as a hairdresser.

## Discussion

Pilonidal sinus is considered to be an acquired disease, irrespective of its localization [5]. It is a condition that most commonly develops in the sacrococcygeal area and, less frequently, in other hair-bearing areas, where an anatomical cleft facilitates the accumulation of hair fragments [6]. Occupational pilonidal sinuses, however, tend to occur in non-hair-bearing areas and do not contain the individual's hair [7]. The disease is mostly observed in hairdressers, but it has also been reported sporadically in other professions, as male sheep shearer, dog groomer or milker of cows [3,7-9].

Pilonidal sinus of the interdigital spaces of the hand is a well-recognised occupational disease of male barbers [4]. The higher incidence of the disease in male hairdressers has been attributed to that female hairdressers seem to be more diligent as concerns the cleansing of the interdigital spaces of the hands and feet [10].

The interdigital spaces are susceptible to penetration by hair because the epidermis is very thin in this area, it is easily irritated by moisturizing agents and shampoos routinely used by hairdressers, while the tile-like formation of the cuticula can act as a barbed hook. Furthermore, clipped hairs are sharp as a needle, moist, electrostatic, adhesive, and preferably accumulate in the web spaces [5]. In our case the papule in the third web space may have also contributed to the formation of the interdigital pilonidal sinus.

The exact reasons for the lesion are not known. However, several theories are offered. Hair penetration, negative pressure from finger abduction, recurrent infection and chronic infection are considered to be factors involved in the establishment of an interdigital web space sinus [11]. The lesion is produced by the penetration of foreign-born short hairs into the interdigital spaces of the hand. The hairs produce an inflammatory reaction and foreign body granuloma [12]. They cause a sinus, and later a cyst. Through the sinus, the hairs get entrapped and may occasionally be expressed [4]. Moreover, chronic, purulent drainage may occur [13]. The structure of the lesions varies from epithelial-lined tract, cyst with surrounding foreign body reaction, to fibrotic cicatricial tissue [12].

Most sinuses are asymptomatic and individuals may not even be aware of them [11,14]. Although the clinical picture is usually benign, it can be complicated by repeated infection, which may require surgery [12]. Abscess formation, cellulitis, lymphangitis and osteomyelitis are possible complications of barber's hair sinus [9,15].

Although thorough removal of imbedded hair might result in complete cure of the condition in certain cases [5], conservative measures in symptomatic sinuses have not actually proved to be totally effective. Despite the fact that surgical excision and skin closure, directly or using a flap, is preferred by many surgeons, it is considered to be prone to recurrence [15]. However, in our case, the wound healed well after primary closure and sutures were removed 7 days after the procedure. Perhaps our case would suggest that thorough curettage might be a contributing factor to successful wound healing by primary intention.

Late recurrence has been reported in the literature and it has been attributed to that, in most cases, patients return in occupation and usually under the same circumstances [14,15].

Pilonidal sinus of the interdigital spaces is a rare and preventable disease. Careful cleansing and drying of the interdigital spaces [5], as well as use of protective barrier creams [7], adhesive band-aid type strips, collodion, or fingerless gloves which maintain pulp sensitivity [8] could prevent the formation of the disease. Moreover, hairdressers are advised to wear socks and shoes that do not expose the feet, in order to prevent the formation of a pilonidal sinus on the feet [5,7]. However, the main preventative method is the careful removal of any hairs that have penetrated the epidermis during the working day [15].

## Consent

Written informed consent was obtained from the patient for publication of this case report and any accompanying images. A copy of the written consent is available for review by the Editor-in-Chief of the «Journal of Medical Case Report»

## Competing interests

The authors declare that they have no competing interests.

## Authors' contributions

All authors contributed the same.
